# Hsp90 modulates human sperm capacitation via the Erk1/2 and p38 MAPK signaling pathways

**DOI:** 10.1186/s12958-021-00723-2

**Published:** 2021-03-04

**Authors:** Peibei Sun, Yayan Wang, Tian Gao, Kun Li, Dongwang Zheng, Ajuan Liu, Ya Ni

**Affiliations:** Department of Reproductive Physiology, Zhejiang Academy of Medical Sciences / Hangzhou Medical College, 310013 Hangzhou, Zhejiang China

**Keywords:** Sperm capacitation, Hsp90, Erk1/2, P38, MAPK signaling pathway, Phosphorylation

## Abstract

**Background:**

Heat shock protein 90 (Hsp90) is a highly abundant eukaryotic molecular chaperone that plays important roles in client protein maturation, protein folding and degradation, and signal transduction. Previously, we found that both Hsp90 and its co-chaperone cell division cycle protein 37 (Cdc37) were expressed in human sperm. Hsp90 is known to be involved in human sperm capacitation via unknown underlying mechanism(s). As Cdc37 was a kinase-specific co-chaperone of Hsp90, Hsp90 may regulate human sperm capacitation via other kinases. It has been reported that two major mitogen-activated protein kinases (MAPKs), extracellular signal-regulated kinase 1/2 (Erk1/2) and p38, are expressed in human sperm in the same locations as Hsp90 and Cdc37. Phosphorylated Erk1/2 has been shown to promote sperm hyperactivated motility and acrosome reaction, while phosphorylated p38 inhibits sperm motility. Therefore, in this study we explored whether Hsp90 modulates human sperm capacitation via the Erk1/2 and p38 MAPK signaling pathways.

**Methods:**

Human sperm was treated with the Hsp90-specific inhibitor 17-allylamino-17-demethoxygeldanamycin (17-AAG) during capacitation. Computer-assisted sperm analyzer (CASA) was used to detect sperm motility and hyperactivation. The sperm acrosome reaction was analyzed by using fluorescein isothiocyanate-conjugated *Pisum sativum* agglutinin (PSA-FITC) staining. The interactions between Hsp90, Cdc37, Erk1/2 and p38 were assessed using co-immunoprecipitation (Co-IP) experiments. Western blotting analysis was used to evaluate the levels of protein expression and phosphorylation.

**Results:**

Human sperm hyperactivation and acrosome reaction were inhibited by 17-AAG, suggesting that Hsp90 is involved in human sperm capacitation. In addition, Co-IP experiments revealed that 17-AAG reduced the interaction between Hsp90 and Cdc37, leading to the dissociation of Erk1/2 from the Hsp90-Cdc37 protein complex. Western blotting analysis revealed that levels of Erk1/2 and its phosphorylated form were subsequently decreased. Decreasing of Hsp90-Cdc37 complex also affected the interaction between Hsp90 and p38. Nevertheless, p38 dissociated from the Hsp90 protein complex and was activated by autophosphorylation.

**Conclusions:**

Taken together, our findings indicate that Hsp90 is involved in human sperm hyperactivation and acrosome reaction. In particular, Hsp90 and its co-chaperone Cdc37 form a protein complex with Erk1/2 and p38 to regulate their kinase activity. These results suggest that Hsp90 regulates human sperm capacitation via the Erk1/2 and p38 MAPK signaling pathways.

## Background

Spermatozoa that have just been ejaculated cannot fertilize oocytes and must undergo a series of biochemical and physiological events in the female reproductive tract to become fertilization competent. This process is known as sperm capacitation [1]. Capacitated sperm usually exhibit protein tyrosine phosphorylation, hyperactivated motility, and acrosome reaction initiation [2], which aids the fusion of sperm into an oocyte. It is generally known that sperm capacitation starts from outflow of cholesterol in the plasma membrane and subsequently increases the permeability of membrane [3]. The increasing influx of Ca^2+^ and HCO_3_^−^ activated soluble adenylate cyclase (sAC) [4–6], which catalyzes the conversion of ATP into cAMP. Subsequently, cAMP activates cAMP-dependent protein kinase A (PKA), which activates target proteins and promotes protein tyrosine phosphorylation [7]. However, the mechanisms that act downstream of PKA during human sperm capacitation remain unclear.

Heat shock protein 90 (Hsp90) is a highly abundant eukaryotic molecular chaperone that plays important roles in various aspects of cell physiology, including client protein maturation, protein folding and degradation, signal transduction, and the assembly of multiprotein complexes [8–10]. Recently, Zierer et al. demonstrated that Hsp90 plays a key role in the activation of various client proteins at the center of cell signaling [11]. Previously, we found that Hsp90 is expressed predominantly in the neck, midpiece, and tail regions of human sperm [12]. Geldanamycin is a specific Hsp90 inhibitor that has been shown to interrupt human sperm capacitation, change protein tyrosine phosphorylation levels, and decrease hyperactivated motility and acrosome reaction. In addition, other studies have found that Hsp90 levels are downregulated in oligoasthenozoospermia and that the functional inhibition of Hsp90 can attenuate progesterone-mediated sperm motility and acrosome reaction [12, 13]. However, the underlying mechanisms remain unknown.

Cell division cycle 37 (Cdc37) is an important kinase-specific co-chaperone of Hsp90 that plays a crucial role in the maturation of numerous kinases by acting as an adapter and loading them onto the Hsp90 complex [14, 15]. In somatic cells, Cdc37 has been shown to bind to kinases during or shortly after translation and recruit them to the Hsp90 protein complex, thereby protecting the kinases from proteasomal degradation and maintaining their activity [16–19]. Recently, we demonstrated that Cdc37 is expressed in the neck and tail regions of human sperm, which also contain Hsp90, suggesting that these proteins may interact to regulate kinases activity during human sperm capacitation [20]. Many kinases regulate sperm function via protein phosphorylation with the help of Hsp90; for instance, PKA and protein kinase C (PKC) phosphorylate Hsp90 in sperm, which regulates non-receptor tyrosine kinase Src during capacitation [20].

Mitogen-activated protein kinases (MAPK) cascades play a pivotal role in signal transduction during many cellular responses, including metazoan development, differentiation, survival, migration, proliferation, growth, and apoptosis [21, 22]. The mammalian MAPK cascade consists of three major tiers of protein kinases, namely MAP kinase kinase kinases (MAP3Ks), MAP kinase kinases (MAP2Ks) and MAPKs, which can be activated in turn to phosphorylate downstream effectors, such as transcription factors, and affect cell differentiation, cell motility, cell death and intracellular trafficking [23]. There are at least three MAPK protein families, including extracellular signal-regulated kinase 1/2 (Erk1/2), p38, and c-Jun N-terminal kinases 1–3 (JNK1-3) [21], and many of proteins in the MAPK pathway interact with Hsp90 [24–26]. Erk1/2 and p38, but not JNK1/2, have been detected in the tail region of mature human sperm [27], consistent with Hsp90 and Cdc37. In addition, many studies have shown that Erk1/2 and p38 are involved in regulating sperm motility: phosphorylated Erk1/2 promotes sperm motility and hyperactivated motility, whereas phosphorylated p38 inhibits sperm motility [28]. Both Erk1/2 and p38 play positive roles in acrosome reaction [29], suggesting that the MAPK pathway may regulating capacitation and acrosome reaction in the female reproductive tract before fertilization.

Since the proteins in MAPK cascades are all kinases, we explored whether Hsp90 and its kinase-specific co-chaperone Cdc37 regulate the MAPK pathway during human sperm capacitation. The findings of this study will help us to understand the mechanisms via which Hsp90 regulates sperm capacitation.

## Materials and methods

### Chemicals and reagents

Percoll was purchased from GE Healthcare BioSciences AB (Uppsala, Sweden). Sodium dodecyl sulfate lysis buffer (used for western blotting, P0013G), cell lysis buffer (used for Co-immunoprecipitation (Co-IP), P0013), and all other reagents for sodium dodecyl sulfate-polyacrylamide gel electrophoresis (SDS-PAGE) and western blotting were acquired from the Beyotime Institution of Biotechnology (Shanghai, China). Protein loading buffer, ECL plus chemiluminescence kit, and pre-dyed protein marker were purchased from Thermo Fisher Scientific Inc. (Burlington, NC, USA). Protein A + G agarose was obtained from Santa Cruz Biotechnology Inc. (Dallas, Texas, USA). Progesterone and fluorescein isothiocyanate-conjugated *Pisum sativum* agglutinin (PSA-FITC) were purchased from Sigma-Aldrich (St Louis, MO, USA). Dimethyl sulfoxide (DMSO) was obtained from Merck (Darmstadt, Germany). 17-allylamino-17-demethoxygeldanamycin (17-AAG) was provided by Cell Signaling Technology (Danvers, MA, USA). Complete Mini EDTA-free Protease Inhibitor Cocktail and Phosphatase Inhibitor Cocktail were obtained from Roche (Mannheim, Germany). Mouse monoclonal anti-Erk1/2 antibody (SC514302), mouse monoclonal anti-p-Erk1/2 (phosphor T202/185 and Y204/187) antibody (SC81492), and anti-Cdc37 antibody (SC13129) were purchased from Santa Cruz Biotechnology Inc.; Rabbit monoclonal anti-p38 antibody (ab170099), rabbit polyclonal anti-p-p38 (phosphor T180 and Y182) antibody (ab4822), rabbit monoclonal anti-Hsp90 antibody (ab203126) and rabbit anti-β-tubulin antibody (ab6046) were purchased from Abcam (Cambridge, UK). Horseradish peroxidase (HRP)-conjugated goat anti-rabbit and goat anti-mouse IgG were purchased from Invitrogen (Carlsbad, CA, USA).

### Human tubal fluid (HTF) medium

HTF medium was prepared as described previously [20]. It was used throughout the study for sperm treatment and consisted of 90 mM NaCl, 5.06 mM KCl, 1.80 mM CaCl_2_, 25.3 mM NaHCO_3_, 1.01 mM MgSO_4_, 1.17 mM KH_2_PO_4_, 5.56 mM glucose, 21.6 mM sodium lactate, 0.27 mM sodium pyruvate, 20 mM Hepes, 4 g/L bovine serum alubm (BSA), 60 mg/L penicillin, and 5 mg/L phenol red. The pH of the medium was adjusted to 7.4 by using sodium hydroxide (NaOH) or hydrochloric acid (HCl). All chemicals mentioned here were provided by Sigma-Aldrich.

### Semen collection and sperm treatment

This study was approved by the Medical Ethics Committee of the Zhejiang Academy of Medical Sciences. Informed consents were obtained prior to semen sample collection form healthy male donors (25 to 35 years old). Fresh semen samples were obtained by masturbation after sexual abstinence for 3–5 days, and liquefied for 1 h at 37°C for subsequent processing. According to World Health Organization (WHO) requirements, sperm samples in this study met the following criteria: sperm motility ≥ 50 %, sperm viability ≥ 85 %, sperm concentration ≥ 20 × 10^6^ sperm/mL, and morphologically normal sperm ≥ 15 %. Semen was centrifuged with 40 % and 80 % discontinuous Percoll gradients at 750 × *g* for 15 min to remove dead sperm and cell debris. The precipitate was resuspended in HTF medium, centrifuged at 500 × *g* for 5 min, and adjusted to a density of approximately 20 × 10^6^ sperm/mL in HTF supplemented with 2.5 µM progesterone. The sperm was divided into aliquots treated with different concentration of 17-AAG (0.5 µM or 5 µM, DMSO as vehicle control), and cultured in a 5 % CO_2_ incubator at 37°C in constant humidity for 3 h.

### Assessment of sperm capacitation

Since only capacitated sperm undergo exocytosis, human sperm capacitation was evaluated indirectly using a progesterone-induced acrosome reaction. According to the WHO Laboratory Manual for the Examination and Processing of Human Semen (5th ed), the acrosome reaction was assessed by PSA-FITC staining. Following capacitation culture, sperm were fixed with 95 % ethanol for 30 min, mounted on Silane-Prep slides, and then air dried and incubated overnight in the dark at 4 ^o^C with 25 mg/L PSA-FITC. Sperm were washed with PBS and examined by fluorescence microscopy (*n* > 200 sperm/sample) (Nikon Eclipse 80i; Nikon Inc., Tokyo, Japan).

### Sperm motility and hyperactivation analysis

Sperm motility and hyperactivation were analyzed using a computer-assisted sperm analyzer (CASA; IVOS, Hamilton-Thorne Bio-Sciences, Beverly, MA, USA) with the following parameters: frame rate, 60 Hz; acquisition frame, 30; minimum contrast, 80; minimum cell size, 3 pixels; cell intensity, 40; path velocity, 25.0 µm/s; straightness threshold, 80 %; slow cell, average path velocity (VAP) and straight line velocity (VSL) of less than 5.0 µm/s and 11 µm/s, respectively; illumination intensity, 2164; magnification, 1.73 ×; temperature, 37 ˚C; and chamber depth, 20 µm (*n >* 200 motile sperm per sample). Briefly, sperm samples (5 µL) were loaded into 20-µm deep of slides chambers warmed to 37 ˚C and the following parameters were assessed: VSL, VAP, curvilinear velocity (VCL), straightness (STR = VSL/VAP × 100), linearity (LIN = VSL/VCL × 100), amplitude of lateral head displacement (ALH), beat-cross frequency (BCF), and the percentage of motile, progressive, and hyperactivated sperm. Sperm hyperactivation was defined using the SORT function as follows: VCL ≥ 150 µm/s; ALH ≥ 7.0 µm; and LIN ≤ 50 %.

### Protein extraction

According to our previously reported method, sperm were washed twice with PBS and resuspended in lysis buffer containing protease inhibitors (Complete Mini EDTA-free Protease Inhibitor Cocktail and Phosphatase Inhibitor Cocktail) and 1 mM phenylmethylsulfonyl fluoride (PMSF). After ultrasonication, the samples were centrifuged at 14,000 × *g* for 20 min and the supernatant was collected. Protein concentration was determined using a bicinchoninic acid (BCA) assay kit (Beyotime Institution of Biotechnology).

### Co-IP experiments

For Co-IP, sperm protein was extracted by ultrasonication in lysis buffer supplemented with 20 mM sodium molybdate (Na_2_MoO_4_), 1 % Nonidet P-40 (NP-40), 1 mM PMSF, Roche Complete Mini EDTA-free Protease Inhibitor Cocktail, and Phosphatase Inhibitor Cocktail. Approximately 500 µg of protein lysate was incubated with 1 µg of rabbit or mouse IgG and 20 µL protein A + G agarose beads under rotation for 2 h at 4 °C before being centrifuged at 1000 × *g* for 5 min. Each of the supernatants were mixed with 2 µg of anti-Cdc37, anti-Erk1/2, or anti-p38 antibodies respectively and then incubated under rotation at 4 ^ο^C overnight. Next, 40 µL of protein A + G agarose beads were added to the mixture and rotated gently for 5 h to capture the antigen-antibody complexes. Precipitates were separated by centrifugation at 1000 × *g* for 5 min and the agarose beads were washed five times with lysis buffer, mixed with 5 × loading buffer, and boiled at 100 °C for 10 min for electrophoresis preparation.

### Western blotting

Equal amounts of sperm protein (20 µg) from different treatment groups or equal agarose beads was denatured by incubation with protein loading buffer at 100°C for 10 min and separated by SDS-PAGE with a pre-stained protein ladder as a molecular weight marker. Proteins were transferred to an Immunoblot-P membrane (Millipore Corporation, Bedford, Massachusetts, USA) which was immersed in 5 % skimmed milk (m/v) in Tris-buffered saline (TBS; pH 7.4) for 1 h at room temperature and incubated with specific antibodies against Erk1/2, phosphor-Erk1/2, p38, phosphor-p38, or Hsp90 at 4°C overnight. After three washes at 5-min intervals with TBS supplemented with 0.01 % Tween-20 (v/v), the membranes were incubated with appropriate secondary antibodies at room temperature for 1 h and washed a further three times. Protein blots were detected using an enhanced chemiluminescence (ECL) kit (Thermo Fisher Scientific) with a gel imaging system (Amersham Imager 600; General Electric Company, USA). As a loading control, membranes were stripped for probing with β-tubulin antibodies. Gray intensity was analyzed using Image J software.

### Statistical analysis

Statistical analyses were performed using the Statistical Package for the Social Sciences software (SPSS, version 23; IBM Corporation, Armonk, NY, USA). Data are expressed as the mean ± standard error of the mean (SEM). One-way analysis of variance was used for statistical analysis. When tests for the homogeneity of variance were significant (*P* < 0.05), data were analyzed using Dunnett’s T3 test; otherwise, the least significant difference test was used. All *P* values were based on two-sided comparisons and values of < 0.05 were considered statistically significant.

## Results

### 17-AAG inhibits human sperm capacitation

**Fig. 1:**
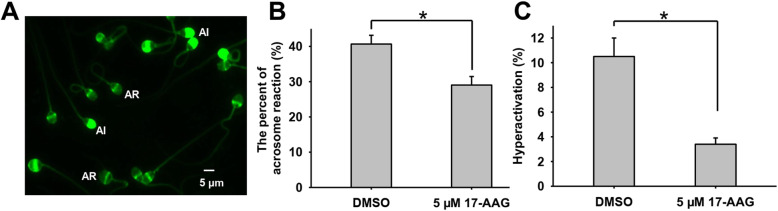
17-AAG inhibits human sperm acrosome reaction and hyperactivation. **a.** The human sperm acrosome reaction was evaluated using PSA-FITC staining, a common method for assessing sperm capacitation based on alterations in sperm head staining. Acrosome reaction (AR) indicates capacitated and acrosome-reacted sperm, while acrosome integrity (AI) indicates uncapacitated sperm. **b**. The acrosome reaction percentage was significantly lower when human sperm were treated with 5 µM 17-AAG than vehicle control (*P < 0.05). **c**. Sperm motility and hyperactivation were analyzed using CASA. The percentage of sperm with hyperactivated motility was significantly lower when treated with 5 µM 17-AAG than vehicle control (*P < 0.05)

Previously, we found that geldanamycin influences human sperm capacitation [12]; therefore, in this study we examined the effect of 17-AAG, a geldanamycin derivative and specific Hsp90 inhibitor, on human sperm capacitation. Since capacitated sperm always display hyperactivated motility and the acrosome reaction, we evaluated the effect of 17-AAG on human sperm capacitation indirectly using acrosome reaction and hyperactivation assays. PSA-FITC staining easily distinguished sperm with an acrosome reaction (AR) and those with acrosome integrity (AI) (Fig. 1a). When human sperm was treated with 5 µM 17-AAG during capacitation, a significantly low percentage underwent the acrosome reaction than when treated with DMSO (vehicle control; Fig. 1b, *P* < 0.05). When sperm motility and hyperactivation were evaluated using CASA, we found that 5 µM 17-AAG markedly decreased sperm hyperactivation (Fig. 1 c, *P* < 0.05). Detailed sperm motility parameters are summarized in Table 1. These results are consistent with our previous findings and suggest that Hsp90 plays a role in human sperm capacitation via unknown underlying mechanisms.

**Table 1:** Effect of 17-AAG on motility parameters in human sperm

	DMSO	5 µM 17-AAG
Motility (%)	62.5 ± 8.5	51.4 ± 2.9
VSL (µm/s)	53.5 ± 4.0	48.6 ± 1.8
VCL (µm/s)	111.3 ± 4.5	93.5 ± 2.4
VAP (µm/s)	64.7 ± 3.7	57.3 ± 1.9
STR (%)	80.8 ± 1.8*	82.2 ± 0.5*
LIN (%)	48.5 ± 1.9	52.0 ± 0.8
ALH (µm)	4.7 ± 0.2	4.1 ± 0.1
BCF (Hz)	28.9 ± 1.2	28.9 ± 0.4
Progressive sperm (%)	39.8 ± 5.8*	33.4 ± 2.6*
Hyperactivation (%)	10.5 ± 1.5*	3.4 ± 0.5*

Results are expressed as means ± standard error of the mean (SEM; *n* = 6). The results showed that 5 µM 17-AAG significantly inhibited human sperm hyperactivation (*P < 0.05). *VSL* straight line velocity; *VCL* curvilinear velocity; *VAP* average path velocity; *STR* straightness (VSL/VAP multiplied by 100); *LIN* linearity (VSL/VCL multiplied by 100); *ALH* amplitude of lateral head displacement; and *BCF* beat-cross frequency

### 17-AAG decreases levels of the Hsp90-Cdc37 protein complex

**Fig. 2:**
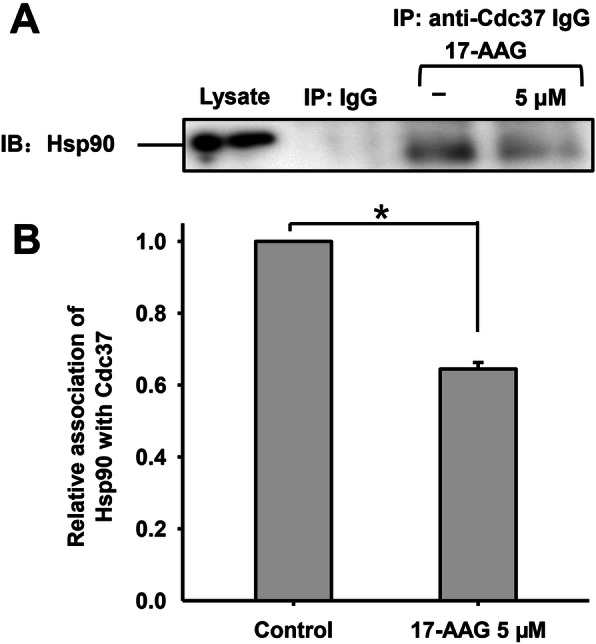
Interaction between Hsp90 and its co-chaperone Cdc37 in human sperm. **a**. Hsp90 was detected in sperm lysates using western blotting and Co-IP with non-immune mouse IgG or antibodies against Cdc37. No interaction was observed between non-immune mouse IgG and Hsp90, whereas a clear interaction was evident between Hsp90 and Cdc37. 17-AAG disturbed the formation of the Hsp90-Cdc37 complex. Results are representative of three independent experiments using samples from three different individuals. **b**. The relative association of Hsp90 and Cdc37 with or without 5 µM 17-AAG during human sperm capacitation

Cdc37 is a kinase-specific Hsp90 co-chaperone that recruits kinases to the Hsp90-Cdc37 protein complex to maintain their activity or stability [30]. To determine whether Hsp90 regulates human sperm capacitation by stabilizing Erk1/2 and p38 or activating their kinase activity, we examined the interaction between Hsp90 and Cdc37 during human sperm capacitation firstly, by treating sperm with or without 5 µM 17-AAG for 3 h, followed by Co-IP. Hsp90 was around 90 kD in size. No interaction was observed between non-immune mouse IgG and Hsp90, whereas a clear interaction was evident between Hsp90 and Cdc37 (Fig. 2a). We also found that the interaction between Hsp90 and Cdc37 was significantly reduced following treatment with 17-AAG. The relative association between Hsp90 with Cdc37 was around 65 % of the control when treated with 5 µM 17-AAG (*P < 0.05) (Fig. 2b), suggesting that levels of the Hsp90-Cdc37 protein complex decreased in the presence of 17-AAG and that kinases bound to Hsp90-Cdc37 may dissociate from the complex.

### 17-AAG causes Erk1/2 to dissociate from Hsp90 and be degraded

**Fig. 3:**
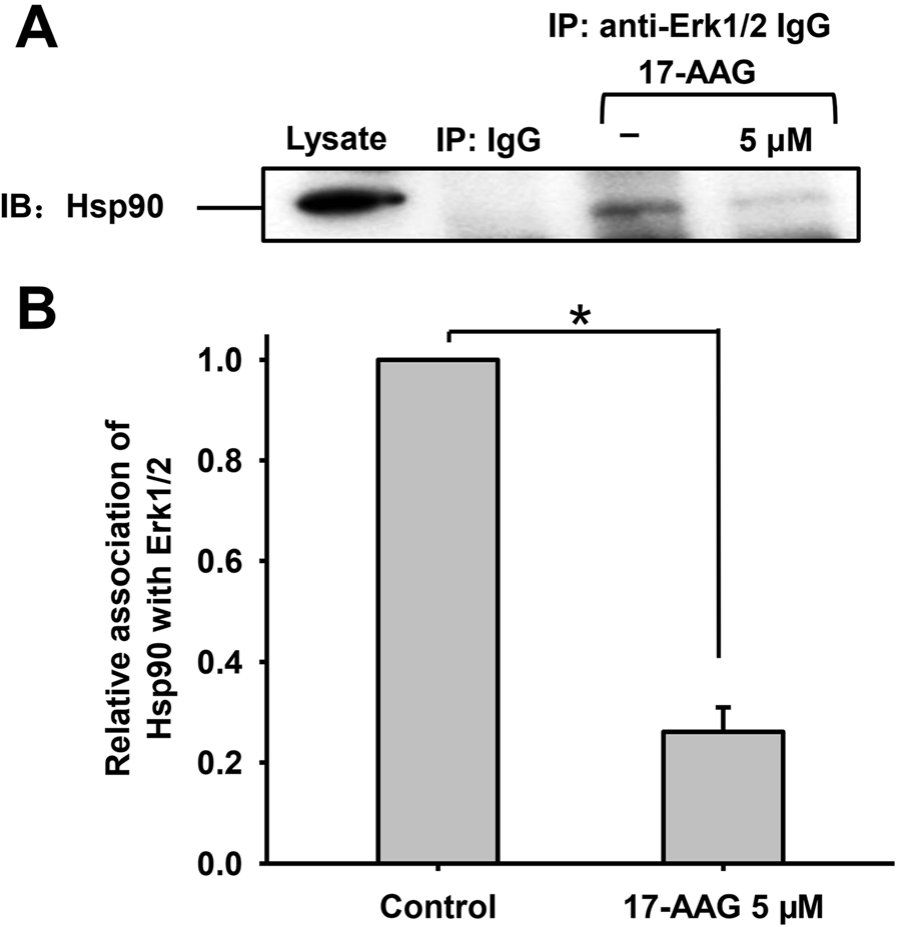
Interaction between Hsp90 and Erk1/2 during human sperm capacitation. **a**. Hsp90 was detected in sperm lysates using western blotting and Co-IP with non-immune mouse IgG or antibodies against Erk1/2. No interaction was observed between non-immune mouse IgG and Hsp90, whereas a clear interaction was evident between Hsp90 and Erk1/2. 17-AAG significantly disrupted the interaction between Hsp90 and Erk1/2. **b**. The relative association of Hsp90 and Erk1/2 with or without 5 µM 17-AAG during human sperm capacitation

Erk1/2 is known to be located in the tail regions of human sperm and regulate capacitation [27]; therefore, we examined the interaction between Hsp90 and Erk1/2 using Co-IP and western blotting. No interaction was observed between the IgG control and Hsp90, yet a clear interaction was evident between Erk1/2 and Hsp90 (Fig. 3a). In addition, 17-AAG significantly decreased the interaction between Hsp90 and Erk1/2. The relative association between Hsp90 with Erk1/2 was around 25 % of the control when treated with 5 µM 17-AAG (*P < 0.05) (Fig. 3b), suggesting that Erk1/2 had dissociated from the Hsp90 complex.

**Fig. 4:**
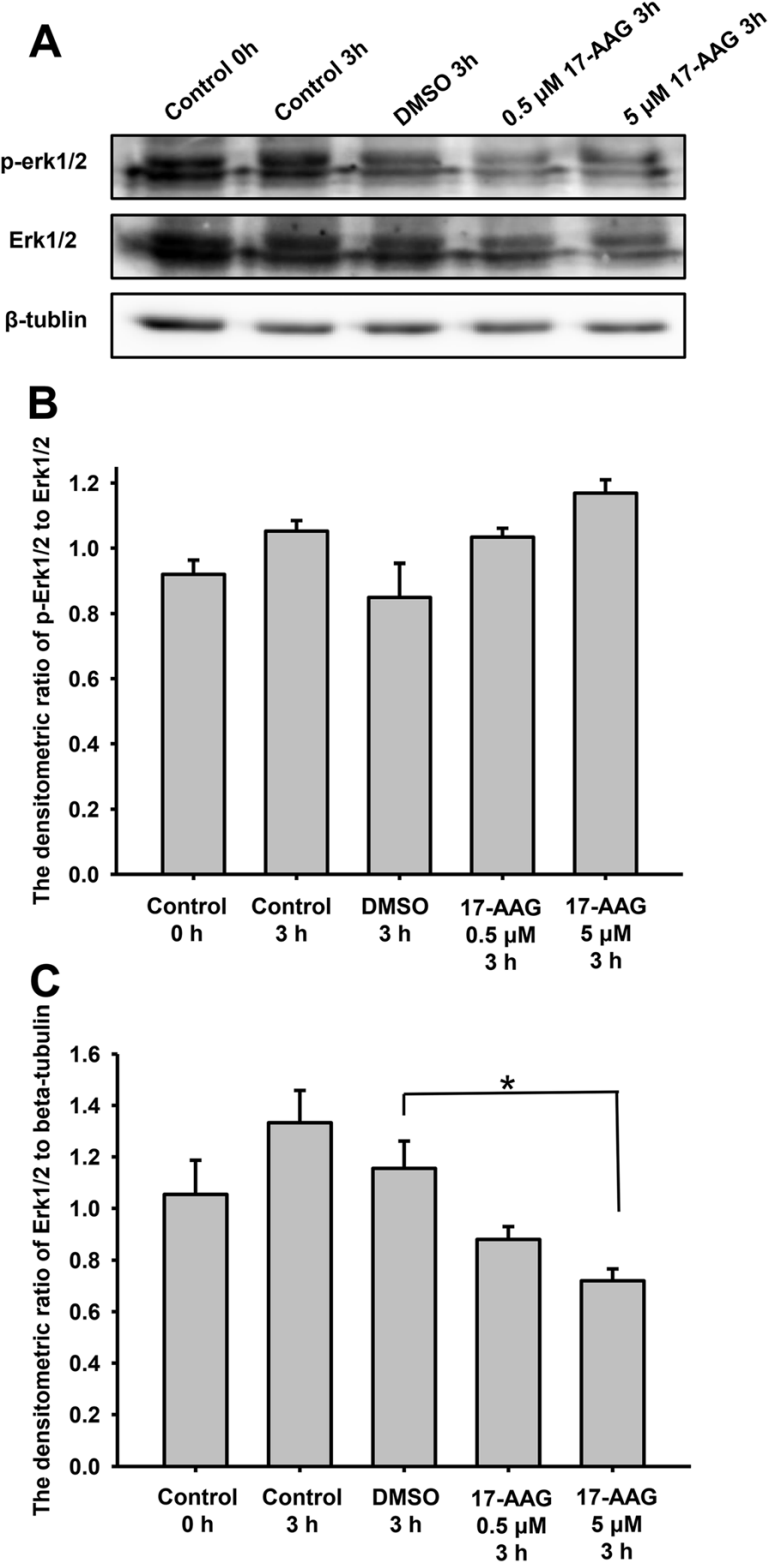
17-AAG affects total and phosphorylated Erk1/2 (p-Erk1/2) expression during human sperm capacitation. **a**. The effects of 17-AAG on p-Erk1/2 and Ekr1/2 were determined by western blotting. **b**. The densitometric ratio of p-Erk1/2 to Erk1/2. No significant difference was observed between the different groups. Data represent the mean ± SEM (*n* = 3) of three independent experiments. **c**. Densitometric ratio of Erk1/2 to β-tubulin. Sperm treated with 17-AAG (5 µM) had significantly lower Erk1/2 expression than vehicle control (*P < 0.05). Data represent the mean ± SEM (*n* = 3) of three independent experiments

Erk1/2 phosphorylation at Thr202/185 and Tyr204/187 is required for enzyme activation [31], and phosphorylated Erk1/2 (p-Erk1/2) has been shown to promote motility, hyperactivated motility, and the acrosome reaction in human sperm. To determine how Hsp90 regulates Erk1/2 function during sperm capacitation, we examined the effects of 17-AAG on Erk1/2 and its phosphorylated form. Sperm were divided into five groups: control 0 h, capacitation for 3 h, treated with DMSO for 3 h (vehicle control), and treated with 0.5 µM or 5 µM 17-AAG for 3 h. After lysis, the proteins were resolved by SDS-PAGE and subjected to western blotting analysis. We found that the level of p-Erk1/2 decreased when treated with 17-AAG (Fig. 4a, upper), as did Erk1/2 expression after 17-AAG treatment for 3 h during capacitation (Fig. 4a, middle). Therefore, we examined the densitometric ratio of p-Erk1/2 to Erk1/2, finding no significant differences between the 17-AAG-treated and control groups (Fig. 4b). However, the densitometric ratio of Erk1/2 to β-tubulin differed significantly between the vehicle control (DMSO) group and sperm treated with 5 µM 17-AAG (Fig. 4 c, *P* < 0.05), suggesting that Erk1/2 was degraded in human sperm treated with 17-AAG.

Together, these findings demonstrate that treating human sperm with 17-AAG during capacitation disrupts the interaction between Hsp90 and Erk1/2. Without the molecular chaperone Hsp90, Erk1/2 may become unstable and be degraded, consistent with the observed decrease in Erk1/2 expression. Since the expression level of Erk1/2 decreased when treated with 17-AAG, the level of p-Erk1/2 decreased; the enzyme activity of Erk1/2 also declined. Since p-Erk1/2 promotes hyperactivated motility and the acrosome reaction, 17-AAG may inhibit human sperm capacitation by interfering with Erk1/2 activity, whereas Hsp90 promotes Erk1/2 stability and kinase activation during capacitation.

### 17-AAG causes p38 to dissociate from Hsp90 and be activated by phosphorylation

**Fig. 5:**
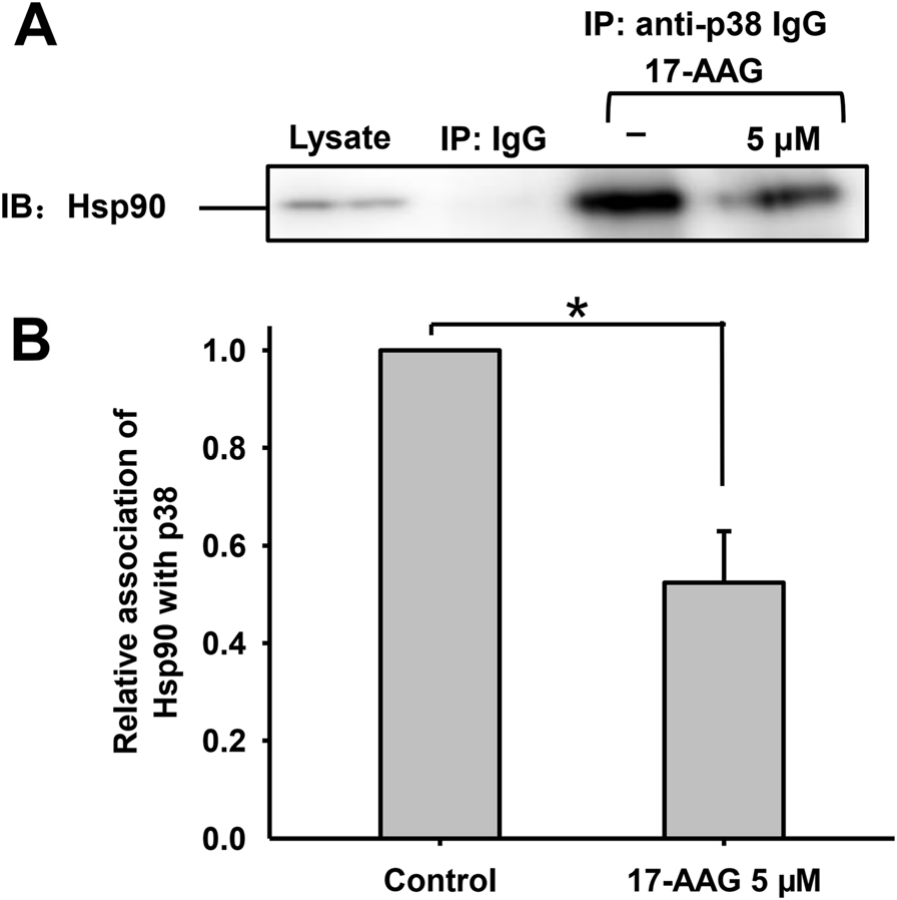
Interaction between Hsp90 and p38 during human sperm capacitation. **a**. Hsp90 was detected in sperm lysates using western blotting and Co-IP with non-immune rabbit IgG or antibodies against p38. No interaction was observed between non-immune rabbit IgG and Hsp90, whereas there was a clear interaction between Hsp90 and p38 that was significantly disrupted by 17-AAG. **b**. The interaction between Hsp90 and p38 with or without 5 µM 17-AAG during human sperm capacitation

Like Erk1/2, p38 is also located in the tail of human sperm alongside Hsp90 and Cdc37 [27]. To elucidate the mechanisms via which Hsp90 regulates p38 during human sperm capacitation, we investigated the interaction between Hsp90 and p38 in sperm treated with 17-AAG (Fig. 5). As expected, there was no interaction between the control IgG and Hsp90; however, Hsp90 interacted closely with p38 during sperm capacitation and the interaction declined significantly when 17-AAG was added. The relative association between Hsp90 and p38 was approximately 45 % of the control when treated with 5 µM 17-AAG (*P < 0.05), suggesting that p38 dissociated from Hsp90.

**Fig. 6:**
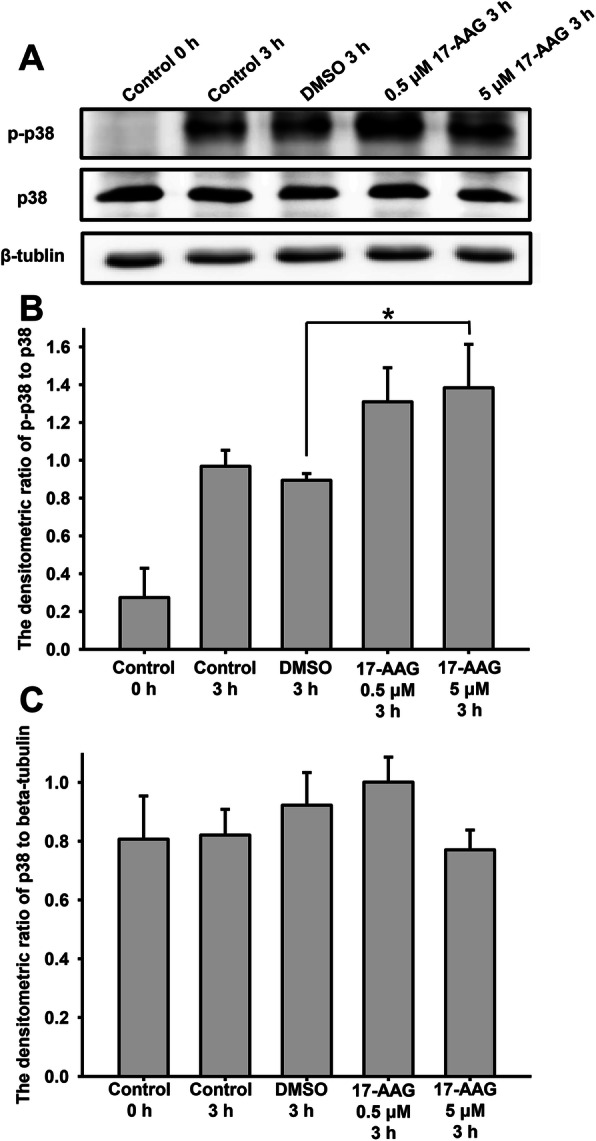
17-AAG affects total and phosphorylated p38 (p-p38) expression during human sperm capacitation. **a**. The effects of 17-AAG on p-p38 and p38 were determined using western blotting. **b**. The densitometric ratio of p-p38 to p38. Sperm treated with 17-AAG (5 µM) had significantly higher p-p38 levels than vehicle control (*P < 0.05). Data represent the mean ± SEM (*n* = 3) of three independent experiments. **c**. Densitometric ratio of p38 to β-tubulin. There was no significant difference between the different groups. Data represent the mean ± SEM (*n* = 3) of three independent experiments

P38 is known to inhibit the forward and hyperactivated motility of sperm and is involved in the acrosome reaction during human sperm capacitation. MAPKs are activated by the phosphorylation of a Thr-X-Tyr motif by upstream kinases; in particular, p38 is activated by phosphorylation at Tyr323 and the subsequent autophosphorylation of Thr180 and Tyr182 [32]. In this study, we examined the effect of 17-AAG on p38 expression and phosphorylation using western blotting. Sperm were divided into five groups: control 0 h, capacitation for 3 h, treated with DMSO for 3 h (vehicle control), and treated with 0.5 µM or 5 µM 17-AAG for 3 h. We finding that phosphorylated-p38 (p-p38) levels increased significantly after capacitation for 3 h, while 17-AAG increased p-p38 levels further (Fig. 6a, upper) but did not change p38 expression (Fig. 6a, middle). Next, we examined the densitometric ratio between p-p38 and p38, finding that p38 was activated after capacitation for 3 h and that 17-AAG promoted p38 activity (Fig. 6b) but not p38 expression (Fig. 6 c).

Together, these results suggest that Hsp90 interacts with p38 during human sperm capacitation by suppressing its phosphorylation and inhibiting its kinase activity. When p38 dissociates from the Hsp90 complex, it is activated by phosphorylation at Thr180 and Tyr182. Since p-p38 inhibits sperm motility and hyperactivated motility during capacitation, the amplification of p38 phosphorylation by 17-AAG may further suppress sperm motility.

## Discussion

**Fig. 7:**
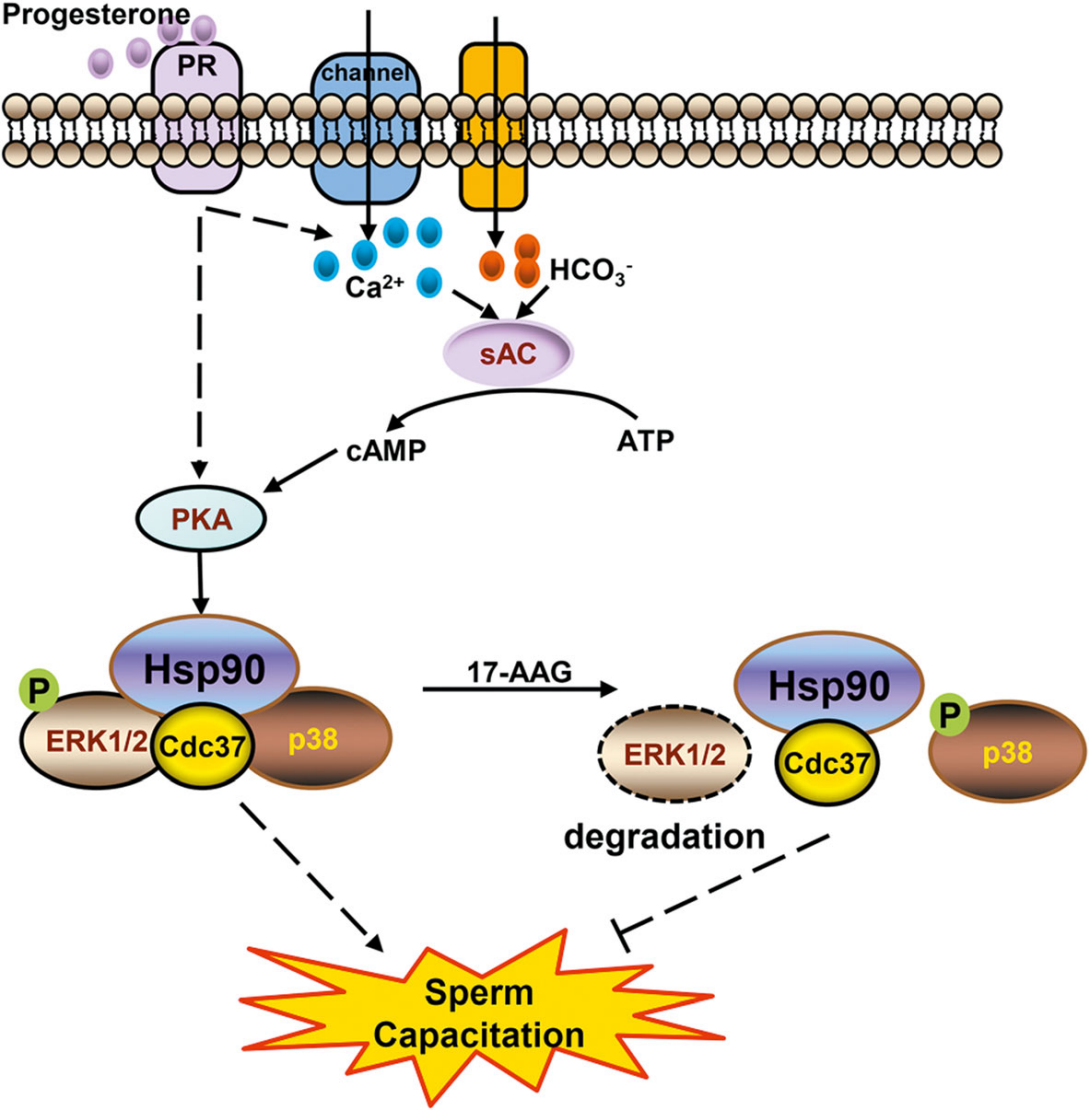
Hsp90 modulates human sperm capacitation via the Erk1/2 and p38 signal pathways During human sperm capacitation, cholesterol outflow from the plasma membrane increases membrane permeability and thus the influx of Ca^2+^ and HCO_3_^−^ activated soluble adenylate cyclase (sAC), which catalyzes the conversion of ATP into cAMP. Subsequently, cAMP activates cAMP-dependent protein kinase A (PKA), which activates target proteins such as Hsp90. Activated Hsp90 and its kinase-specific co-chaperone Cdc37 form a protein complex with Erk1/2, which stabilizes Erk1/2 and maintains its phosphorylation. Phosphorylated Erk1/2 is activated and promotes sperm hyperactivation and the acrosome reaction. Hsp90 and Cdc37 also form a protein complex with p38, which maintains unphosphorylated p38. Since phosphorylated p38 is activated and inhibits sperm hyperactivation, the Hsp90-Cdc37-Erk1/2-p38 complex promotes human sperm capacitation. Treatment with 17-AAG, an Hsp90 specific inhibitor, causes Erk1/2 and p38 to dissociate from the complex. Erk1/2 is subsequently degraded and dephosphorylated, whereas p38 activates itself via autophosphorylation. Ultimately, these changes inhibit human sperm capacitation

Capacitation is the process that sperm undergo a series of biochemical and physiological events in the female reproductive tract to become able to fertilize an oocyte. It is a prerequisite for human sperm fertilization [31, 33]. The mechanisms of capacitation are complicated, which encompass the outflow of cholesterol, regulation of ion permeability and protein phosphorylation. After capacitation, sperm appear hyperactivation and acrosome reaction, which aids the fusion of sperm into an oocyte. Ca^2+^ is essential to regulate human sperm capacitation, sperm motility and acrosome reaction. At the initial stage of capacitation, intracellular increasing of Ca^2+^ and HCO_3_^−^ activate soluble adenylate cyclase (sAC), which catalyzes ATP into cAMP; cAMP subsequently activates PKA, which activates target proteins, promotes protein tyrosine phosphorylation and induces capacitation (Fig. 7) [7, 31, 34]. However, the mechanisms that act downstream of PKA during human sperm capacitation remain unclear.

Hsp90 is expressed ubiquitously in various cell types and plays critical roles by interacting with client proteins [8]. Previously, we found that Hsp90 is expressed in human sperm and is mainly localized in the neck and tail regions. By using geldanamycin, an Hsp90 specific inhibitor, we have demonstrated that Hsp90 is involved in intracellular calcium homeostasis, regulating protein tyrosine phosphorylation, sperm hyperactivated motility, and the acrosome reaction in response to progesterone [12]. Here human sperm were treated with geldanamycin derivative 17-AAG (another Hsp90 specific inhibitor) during sperm capacitation, while the procedures for sperm capacitation culture were accordance with our previous work. Consistently, we found that 17-AAG also influences the acrosome reaction and hyperactivation of human sperm, suggesting that Hsp90 regulates human sperm capacitation. However, the mechanisms still unknown.

It was previously reported that around 60 % of the human kinome interacts with Hsp90 [33], with the assistance of its kinase-specific co-chaperone, Cdc37, which promotes kinase maturation by acting as an adapter and recruiting them to the Hsp90 protein complex. In many tumor cells, Cdc37 overexpression is oncogenic [15], as it mediates the recruitment of kinases to the Hsp90 system and maintains their enzyme activity. Recently, we found that Cdc37 is also expressed in human sperm in the same location as Hsp90. Hsp90 interacts with Cdc37 and is phosphorylated by PKA during human sperm capacitation [20]; therefore, we hypothesized that Hsp90 may further regulate human sperm capacitation via important kinase pathways downstream PKA with the help of Cdc37.

In this study, we demonstrated that Hsp90 and Cdc37 interact with each other during human sperm capacitation. The interaction between Hsp90 and Cdc37 is mediated by binding of the Hsp90 middle domain to an N-terminal region of Cdc37 [14, 35]. When human sperm was treated with 17-AAG, a benzoquinone ansamycin antibiotic that specifically targets Hsp90 and interferes with its function as a molecular chaperone, levels of the Hsp90-Cdc37 protein complex decreased significantly (~ 65 % compared to the control) (Fig. 2). Previous studies found that 17-AAG and its analog, geldanamycin, did not disrupt the interaction between Hsp90 and Cdc37 [36, 37]; however, we observed a decrease in Hsp90 levels in the immunoprecipitated Cdc37 complex treated with 17-AAG. Previously, we showed that geldanamycin decreased Hsp90 expression in sperm in a dose-dependent manner [12], while other studies have found that 17-AAG downregulates Hsp90 expression at the post-translational level in bladder cancer cells [38]. Since 17-AAG is a geldanamycin derivative with an allylamino group at position 17 of the scaffold structure and a similar biological action [38], 17-AAG may reduce Hsp90-Cdc37 levels by decreasing Hsp90 expression in human sperm, thus explaining this discrepancy. Together, these findings suggest that 17-AAG reduces levels of the Hsp90-Cdc37 protein complex during human sperm capacitation, thereby affecting kinases recruited to Hsp90-Cdc37 via Cdc37.

We also found that 17-AAG significantly interrupted the interaction between Hsp90 and Erk1/2 in human sperm (~ 25 % compared to the control) (Fig. 3) and led to greater dissociation than between Hsp90 and Cdc37. These findings suggest that the decrease observed for the Hsp90-Erk1/2 complex was caused by both a decrease in Hsp90 expression and a disrupted interaction between Hsp90 and Erk1/2. Eckl et al. also observed that Erk2 interacts with the Hsp90-Cdc37 complex [14], which was accordance with our findings.

Following 17-AAG administration in human sperm, Erk1/2 dissociated from the Hsp90 complex and was degraded, thereby reducing Erk1/2 activation (p-Erk1/2). Studies in human urinary bladder cancer cells have also reported that total and phosphorylated Erk1/2 protein levels are reduced in a 17-AAG-dependent manner [38]; however, the levels of total Erk1/2 following 17-AAG treatment differed depending on the cell type or cell line [38–40]. Consequently, the decrease in Erk1/2 levels may depend on cell type, 17-AAG concentration, and incubation time. In this study, Hsp90 promoted the stability and activity of Erk1/2 in human sperm, while previous reports have suggested that Erk1/2 phosphorylation promotes sperm motility and acrosome reactions [28]. Therefore, 17-AAG may inhibit human sperm capacitation by disrupting the Hsp90-Cdc37-Erk1/2 complex and affecting Erk1/2 stability, thereby decreasing the levels of active phosphorylated Erk1/2.

The interaction between Hsp90 and p38 also decreased following 17-AAG administration during human sperm capacitation; however, when p38 dissociated from the Hsp90-Cdc37 complex, its phosphorylation at Thr180 and Tyr182 increased. Previously, Ota et al. demonstrated that p38 interacts with Cdc37 and is thereby recruited to the Hsp90-Cdc37 complex in cardiomyocytes; Hsp90 inhibition resulted in the dissociation of p38 from the Hsp90 complex and its subsequent activation via autophosphorylation [26]. In osteoblasts, geldanamycin and 17-AAG have been shown to significantly enhance phosphorylated p38 levels in a dose-dependent manner [41]. Collectively, these results support the possibility that 17-AAG treatment causes p38 to dissociate from the Hsp90-Cdc37 complex and activate itself via autophosphorylation in human sperm. In addition, p-p38 has been shown to inhibit sperm motility, which may explain why 17-AAG inhibited hyperactivated motility in human sperm. Although p-p38 can also promote the acrosome reaction, 17-AAG ultimately inhibited human sperm capacitation.

## Conclusions

In summary, the findings of this study suggest that the specific Hsp90 inhibitor, 17-AAG, influences human sperm capacitation by disrupting the interaction between Hsp90-Cdc37 and the MAPK kinases Erk1/2 and p38. In particular, 17-AAG inhibited capacitation by decreasing total and p-Erk1/2 levels and increasing p-p38 levels (Fig. 7). To our knowledge, this is the first study to report that Hsp90 regulates human sperm capacitation via the Erk1/2 and p38 MAPK signaling pathways.

## Data Availability

All data used during the study are available from the corresponding author by request.

## Abbreviations

Hsp90: Heat shock protein 90
Cdc37: Cell division cycle protein 37
MAPKs: Mitogen-activated protein kinases
Erk1/2: Extracellular signal-regulated kinase 1/2
17-AAG: 17-allylamino-17-demethoxygeldanamycin
PSA-FITC: Fluorescein isothiocyanate-conjugated Pisum sativum agglutinin
sAC: Soluble adenylate cyclase
PKA: Protein kinase A
PKC: Protein kinase C
MAP3Ks: MAP kinase kinase kinases
MAP2Ks: MAP kinase kinases
JNK1-3: c-Jun N-terminal kinases 1–3
